# Immunological Nuances and Complications of Pediatric Organ Transplant: A Narrative Review

**DOI:** 10.7759/cureus.46309

**Published:** 2023-10-01

**Authors:** Vivek R Velagala, Namrata R Velagala, Arihant Singh, Tanishq Kumar, Swedaj Thakre, Yashwant Lamture

**Affiliations:** 1 Medicine, Jawaharlal Nehru Medical College, Datta Meghe Institute of Higher Education and Research, Wardha, IND; 2 Surgery, Jawaharlal Nehru Medical College, Datta Meghe Institute of Higher Education and Research, Wardha, IND

**Keywords:** anti-human leukocyte antigen antibodies, immunological response, graft rejection, acute graft vs host disease, post transplant lymphoproliferative disorder, post-surgical infections, solid organ transplant

## Abstract

Organ transplantation is considered an exaggerated immune state in which the body reacts in an elaborate cascade of reactions against the lifesaving graft transplanted. Unrepairable organ damage is the main indication for a pediatric patient to undergo a transplant. The host and the donor must fulfill the criteria for a successful transplant to have as few side effects as possible. There has been much-needed research in the domain of surgery of organ transplantation, thereby extending into the pediatric age group. This article elaborates on the post-transplant management, the immuno-biochemistry aspect, and its post-surgery treatment. The post-surgery period requires great emphasis as morbidity and mortality are highest. There is much to understand about managing transplant patients to avoid complications such as infections, hypertension, or side effects of immunosuppressive drugs. The treating clinician faces the challenges of managing the dose and frequency of immuno-suppressive medicines to prevent complications in the patients. If the dose is inadequate, there are chances of graft rejection. If the immuno-suppression is prolonged, there may be chances of infections in the patient. This article aims to summarize the mechanism of graft rejection and put forth the need for further research about creating a universal protocol for managing a patient's immune system post-transplant. The authors hope this protocol will help the clinician better understand the patient's current state and help in appropriately using immuno-suppressive drugs. It calls upon the need for a reliable and easily repeatable battery of investigations that will help solve this dilemma.

## Introduction and background

**Figure 1:**
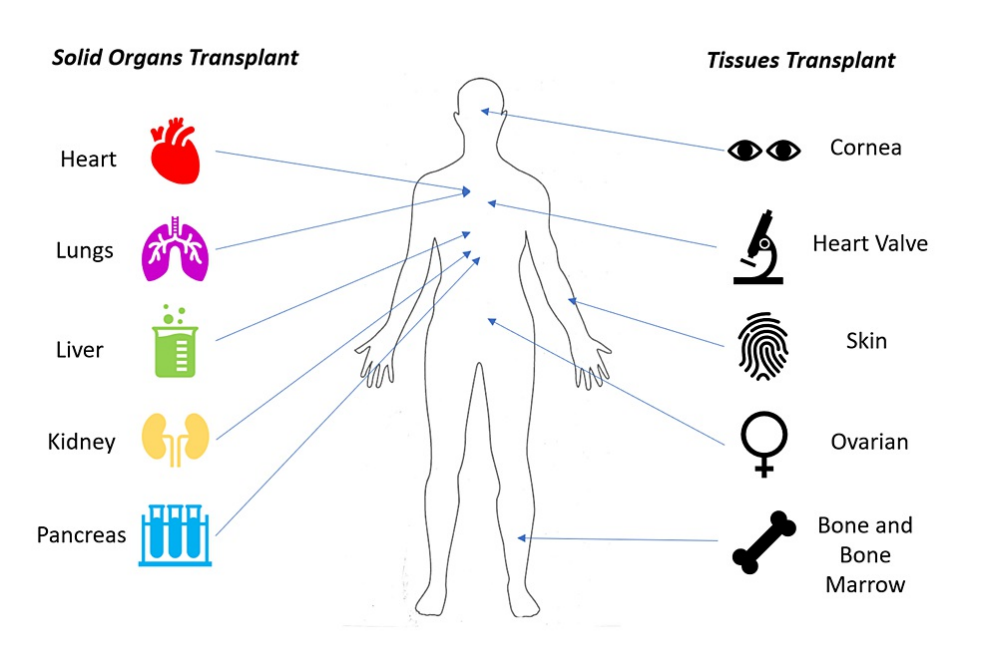
Different organs and tissues that can be transplanted. The figure is the author's own creation.

Organ transplantation is the surgical process involving organ transfer from a donor to a recipient. This transplantation can be in terms of solid organ transplantation or tissue transplantation [1]. Tissue transplantation includes hematopoietic stem cells from bone marrow, bone, cornea, heart valves, and skin [2-4]. In a solid organ transplant (SOT), one or multiple organs are transferred to a patient needing those corresponding organs. These organ transplants are lifesaving procedures indicated when the organ's function has completely diminished or has reached a point of no return. Such organs are the heart, lungs, liver, kidneys, or pancreas [5,6]. The tissue and organs of human transplantation are depicted in Figure 1.

Extensive developments have been made in the surgical part of organ transplantation, and similar depth is also required to manage the patient post-transplant. This management pivots around the immunological responses and cascade of reactions that affect the overall immunochemistry of the patient [7]. The body possesses the innate ability to differentiate between self and non-self. This power of the body is kept under check for the transplantation to be successful [8]. Human Leukocyte Antigen (HLA) is almost unique in every individual, and a match between the donor and the patient plays a vital role in this mechanism. The better the match, the higher the chances for graft acceptance [5]. The primary goal of the management of transplantation is to increase graft survival and avoid graft rejection. Pediatric organ transplant is one such topic under this umbrella, encompassing its unique challenges and management.

## Review

Immuno-suppression drugs

Immuno-suppression is the focus of the treatment to prevent graft rejection due to the hyperactivity of the recipient's immune cells. All immuno-suppressants have a varied spectrum of drug-to-drug interactions. The maximum efficacy of the drugs can be obtained through therapeutic drug monitoring [9]. The mainstay immuno-suppression drugs are calcineurin inhibitors, namely tacrolimus and cyclosporin [10]. They inhibit calcineurin, a calcium and calmodulin-dependent protein phosphatase. Calcineurin is a serine/threonine protein phosphatase that participates in the calcium-dependent T-cell activation pathway [11]. Calcineurin inhibitors are regulated through the induction and inhibition of cytochrome P450 CYP3A enzymes, influencing the effect of immuno-suppression on the patient [9,10,12,13]. Such regulation also has increased side effects, possibly leading to graft rejection. Hence, to prevent graft rejection, anti-proliferative agents are also administered. These are used with corticosteroids, glucocorticoids, rapamycin, and anti-proliferative drugs like mycophenolic acid and azathioprine. Every treatment plan after the transplant aims to strike a balance between avoiding signs of graft rejection and controlling the side effects [12]. A few articles state contradictory views, stating tacrolimus is preferred over cyclosporin as the first-line calcineurin inhibitor due to its increased efficacy and less nephrotoxicity. On the contrasting side, a few articles considered the effect of tacrolimus and cyclosporin as almost the same, with similar incidence and risk of developing side effects such as nephrotoxicity and neurotoxicity [12,14]. Neurotoxicity is presented as headaches, seizures, and tremors. Calcineurin inhibitors cause vasoconstrictive effects, especially in the glomerular vessels, leading to nephrotoxicity. Vasoconstriction is accompanied by hypertension and adds to the nephrotoxicity. Hypertension is controlled with the help of additional anti-hypertensive drugs such as calcium channel blockers [13,14]. They not only bring hypertension under control but also help reduce nephrotoxicity. Calcineurin inhibitors precipitate an imbalance of sodium-related levels and are used in kidney organ recipients. On the other hand, it should be given under monitoring for liver organ recipients [13].

Other commonly reported side effects are hyperlipidemia, gout, and glucose intolerance. The treatment plan gets altered according to the patient's already pre-existing morbidities, such as diabetes [15]. The onset and presentation of the side effects in each individual are different due to polymorphic genetic composition and variables in non-modifiable factors. Bringing yet another layer as to why striking the right balance between effects and side effects is essential. Required modifications can be made to the dosage to create a more personalized and inclusive to how receptive the patient is to the drug's effects [16]. With the development of these newer immunosuppressive drugs, there has been better control over adverse outcomes such as acute rejection. However, there has been an increase in the frequency of the side effects and overall compliance with the treatment regimen [17]. An extensive systematic review by Tsampalieros et al. concluded that the withdrawal or avoidance of the steroids would lead to an increase in the height of prepubertal patients at the same time and would not decrease graft survival.

Infectious complication of immuno-suppression

The most notorious complication following the organ transplant is the risk of infection and development of sepsis, especially in the pediatric age group, which is already a high-risk group for infectious diseases. The risk of sepsis is highest in the first 180 days of life [18]. Infections increase the chances of graft rejection. There are three periods, the first being the period of post-transplantation (less than four weeks). Subsequent days following comprise the period of maximum immunosuppression (four weeks to twelve months) and, finally, the period after twelve months [19,20]. Following the transplant, the most significant risk is transferring disease from the donor to the recipient, recipient-derived, or nosocomial infection leading to sepsis. In the period of maximum immuno-suppression, the risk shifts to the development of opportunistic infections and is greatly influenced by the drugs used in prophylactic treatment. During the later days, the risk lies in community-acquired infections [19,21]. Identifying the phase the patient is in would help the clinician understand the patient's immunosuppressive status.

Bacterial and Viral Infections

Bacteria that commonly cause infection in this state include Staphylococci, Gram-negative bacilli, Listeria, Nocardia, and *Mycobacterium tuberculosis*. In the same studies, these bacterial infections are diagnosed based on clinical symptoms, cerebrospinal fluid findings, brain imaging, blood samples, and microbiological culture [19,22]. *Clostridium difficile *infection is notorious as it can occur in 3-16% of kidney transplant patients with high recurrence rates. Their management is done by giving corresponding antibiotics that do not cause drug-to-drug interactions [21,23].

**Figure 2:**
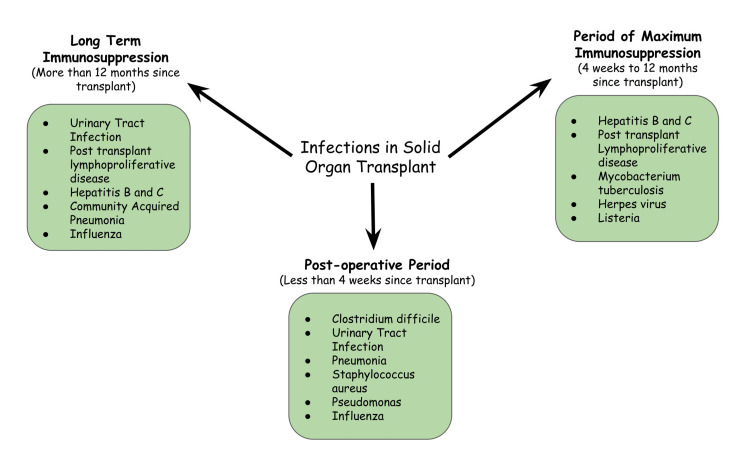
Infections in solid organ transplant. The figure is the author's own creation.

Viral etiologies include Herpes simplex, varicella zoster, Epstein-Barr, and damaging of the lot, cytomegalovirus (CMV). Cytomegalovirus has been proven to cause immuno-suppression, leaving the recipient more prone to further secondary infections. Using lymphocyte-depleting agents such as anti-thymocyte globulin to prevent graft rejection leaves the patient more prone to CMV infections. Another treatment modality is rituximab, a monoclonal antibody against CD20 of B-cells. Use of rituximab on chronic use is shown to cause an increase in incidences of recurrent infection [24,25]. Prophylactic measures against the same include drugs like acyclovir, ganciclovir, and IgG antibodies [24]. Epstein-Barr virus (EBV) is another herpes virus that initially remains asymptomatic and latent, and its manifestations start post-transplant. It may present as asymptomatic viremia or as infectious mononucleosis. However, the most concerning is post-transplant lymphoproliferative disease (PTLD); it is one of the most significant factors leading to the consequence of graft rejection. Its management becomes imperative to save the transplant. It is treated with immunosuppression reduction [21,26]. Another approach to managing EBV is that the immune-suppressive drugs are not stopped but are continued even during the risk of sepsis. This approach is done by carefully monitoring additional medications to control the infection. These drugs are chosen in a way so they do not cause drug-drug interactions. However, immuno-suppressive prescriptions should be stopped in critically ill patients to accelerate their recovery. Such a situation occurs when the patient shows symptoms of active EBV infection [22,27]. The various infections according to the time phases after transplantation have been depicted in Figure 2.

There is a need to control the risk factor for infections at each stage, be it intra-operative complication (bleeding, ischemia, thrombosis, or prolonged operation time), pre-transplant infection in the donor and the recipient, or post-transplant. Recent studies have shown that weaning medications after pediatric organ transplants are safe. There is much scope for further research in creating a standardized immuno-suppression and prophylaxis [22]. Repeated infections can create life-threatening complications and accelerate the degeneration of the graft transplanted, reducing graft survival years.

Immunization of transplant patients

The transplant can occur at any age and hence may affect the overall immunization of the child to vaccine-preventable diseases. There is a dire need for further research on vaccinating children who have undergone transplantation. Such a modality is required to decrease hospitalizations for infections that vaccines can prevent. This under-immunization is due to the failure to generate an adequate response to the vaccine in transplant patients compared to normal individuals. The immuno-suppressive drugs bring about this effect. By general rule, the higher the dose of immune-suppression medications, the lesser the immune response generated to the vaccine. The mechanism of vaccines relies on the generation of immunological reactions and the formation of memory B cells. When no immunological response is developed on vaccine administration, there will be short-lived immunity. The immune system is already compromised at birth and matures through actively and passively acquired immunity. The child develops such immunological maturity as the child grows up and has repeated environmental exposure. The advent of vaccinations has sped up the process. It brings a new dilemma of creating the necessary amount of immunological triggers to create sufficient prolonged immunity in a child who has undergone transplantation and is currently under immunosuppressive drugs. Suppose the child undergoes a transplant when under the age of two years. In that case, there is incomplete immune system maturation and, hence, has been shown to have higher incidences of hospitalization [28]. Another study highlights the importance of herd immunity to be attained as a goal of immunization to help alleviate the situation and provide protection to those without adequate immunity. Obstacles to herd immunity include lack of awareness, vaccine hesitancy, and refusal [29]. Hence, these studies emphasize getting the age-appropriate vaccination done prior and the rest of the immunization done at earlier dates before the transplant [30,31].

Graft rejection

The goal following a transplant is to increase the graft survival as much as possible to allow stable growth and well-being of the person. Maximizing this is essential in the pediatric age group with the highest expected life years [32]. There are two pathways via which graft injuries can occur in a recipient, and the first is antibody-mediated immune responses to the human leukocyte antigen (HLA). The other less researched pathway is via non-HLA antibodies mediated graft rejection [32,33]. The presence of circulating anti-HLA indicates antibody-mediated rejection (AMR) and hence helps to differentiate cellular-mediated rejection (CMR) [33]. A study by Butts et al. showed that HLA matching of more than or equal to three loci is associated with fewer incidences of vasculopathy hence lesser acute graft rejection risk [34]. T cells bring about both CMR and help differentiate B cells, leading to AMR. This mechanism is prevented following the transplant to avoid the formation of donor-specific antibodies (DSA) [35,36]. Studies also show the deposition of C4D deposits in the transplanted kidneys, which was associated with AMR eventually, acute rejection [37,38]. There is also evidence of C4d positive in the liver and anti-HLA antibodies [38]. Rejection also depends upon the involved organ's tissue mass. More mass would correspond to increased tolerance, especially in liver transplant patients. Increased tolerance translates to the ability of the organ to combat the immunological assault [39].

T-Cell and B-Cell Immunomodulation

T cell-mediated allorecognition, as the name suggests, is the identification of the HLA of the donor on the organ by the recipient T cells. Two significant processes regulate it; these pathways involve the T cell receptors (TCR) [33]. There is direct recognition or the indirect process via antigen-presenting cells. The direct process confers to the earliest adaptation steps to the foreign tissue. The pivotal role is the signal of TCR recognition followed by the second signal of immune checkpoints such as CD28, which stimulate T cells [40]. B cells confer humoral immunity and are activated by recognizing the B-cell receptor (BCR), initiating a cascade. Activated B cells form antibodies and confer memory to the immune system. Hence, it can be highly detrimental if they proliferate without proper control. Rituximab is targeted against CD20 on the B cell surface and is therefore used in desensitization [41].

Gulleroglu et al. proposed treatment options in antibody-mediated rejection, comprising rituximab, intravenous immunoglobulins, and plasmapheresis. The same study also elaborates that these modalities can only control and resolve the crisis at hand but not the problem, as there is a remaining risk of chronic antibody-mediated rejection and decreased life span of the graft, especially in pediatric patients [25,42,43]. The earliest manifestation of graft failure is using the gold standard investigation of tissue biopsy. The problem arises with the feasibility of biopsy extraction. It is too invasive and cannot be used for monitoring. Clinically, graft failure can only be established after evidence of organ damage. The presence of antibodies is not a reliable marker to diagnose graft dysfunction [33,44]. Richmann et al. used cardiovascular magnetic resonance as one such investigation. The research also highlighted the requirement to develop reliable and repeatable investigations to detect and monitor transplant organ function [44].

Post-transplant lymphoproliferative disease

Within two years of transplant, there is a high risk of developing lymphoproliferative disorders in EBV serostatus-positive children at the time of the transplant. Post-transplant lymphoproliferative disease (PTLD) includes neoplasms such as Hodgkin lymphoma to benign proliferation and infectious mononucleosis syndrome. It has a low incidence of 1-3% but has a poor prognosis and requires early detection or anticipation. The proliferation usually occurs in the T-cell immunosuppression phase, where cytotoxic T-cell function is reduced. This suppression manifests as an uncontrolled proliferation of infected B cells [45]. Hence, radiation and surgical therapies are added to the usual line of treatment of rituximab, intravenous immunoglobulins, and plasmapheresis [46]. However, one of the significant changes in treatment patterns is the decrease of immunosuppression with a reduced risk of acute rejection. There is much ongoing work on the withdrawal of steroids used for immunosuppression.

## Conclusions

Immunological maturity is attained as the child grows and is exposed to environmental factors, and hence the development of immunity in pediatric organ transplant patients is influenced by the advent of vaccination and the use of immuno-suppressive drugs. There is still a gap in how to manage a patient who has undergone a transplant in the first few days of life and what is such a patient's morbidity index. Infancy might also be considered the best time for an organ transplant as the immune system is not yet mature enough to recognize and reject the transplant effectively. However, there is also the toll it takes on the child, and it may have further consequences in the maturation of the immune system. The immune system's lack of development will precipitate an increased frequency of infections after the transplant and it becomes imperative to keep a state of readiness and anticipation on how to deal with such conditions. This article attempts to be a catalyst by highlighting the domains of much-needed research in developing an investigation that can be done to understand the patient's immunological state at that given time and to prevent complications, reducing morbidity and mortality in the pediatric age group.
